# Metals-induced functional stress in sulphate-reducing thermophiles

**DOI:** 10.1007/s13205-015-0342-1

**Published:** 2016-01-09

**Authors:** Ali Hussain, Javed Iqbal Qazi

**Affiliations:** 1 Department of Wildlife & Ecology, University of Veterinary and Animal Sciences, Lahore, Pakistan; 2 Department of Zoology, University of the Punjab, Lahore, Pakistan

**Keywords:** Bioprecipitation, Economical bioremediation, Metallic pollutants, Sulphate-reducing bacteria, Thermophiles

## Abstract

All toxic metals have been known to inhibit different activities of sulphate-reducing bacteria (SRB) at different concentrations. The present study delineates functional responses of two thermophilic SRB species (*Desulfotomaculum reducens*-HA1 and *Desulfotomaculum hydrothermale*-HA2) to toxic metals. Bacterial activity was assessed in terms of sulphate reduction and metal precipitation employing four concentrations (1, 5, 10 and 15 ppm) of three dissolved toxic metals (Cu, Cr and Ni) independently. Both sulphidogenic bacterial species showed results in a very narrow range of fluctuations. In general, bioprecipitation and sulphate reduction were pronounced at lower concentrations (1 and 5 ppm) and got inhibited at higher concentrations (10 and 15 ppm). The order of precipitation and sulphate reduction for the subject metals was Ni > Cr > Cu. The findings of this study will be helpful in developing economical and environmental friendly bioremediation process(es) tending to operate at extreme conditions around the concentrations in indicated suitable metals-loaded effluents.

## Introduction

A plenty of multidimensional anthropogenic activities especially associated with mining and other industrial processes are introducing metals continuously into the environment. Direct and indirect deleterious effects of metal ions to the environment and human health are well known (Goyer and Clarkson 2001; Landis and Yu 2004; Kucukatay et al. 2007; Mortazavi and Jafari-Javid 2009; Becker et al. 2010; Si et al. 2015). Therefore, treatment of these metallic pollutants is much necessary before discharging them to the environment. The most widely used method for the removal of metals’ ions is chemical neutralization and being expensive and possible generation of secondary pollutants, its application is question marked (Saeed and Iqbal 2003; Rocha et al. 2009). Biological treatment of metal-loaded effluents is an attractive alternative. The major advantages of the biological methods are their better performance, low operational cost, minimum generation of secondary pollutants and environmentally compatible natures (Malik 2004; Okoh and Trejo-Hernandez 2006). Implication of dissimilatory sulphate-reducing bacteria (DSRB) in detoxifying metal contaminated environments has gained importance among all the biological methods practiced for the removal of metals in the last decade (Neculita et al. 2007; Martins et al. 2009; Alexandrino et al. 2011; Barbosa et al. 2014).

SRB make morphologically and physiologically a diverse group of strict anaerobes. Under anaerobic conditions, they dissimilate sulphate to sulphide while utilizing various types of low molecular weight substrates (including various environmental contaminants) as source of carbon and energy (Willis et al. 1997; Hussain and Qazi 2012, 2014; Hussain et al. 2014a, b). The biogenic sulphide, thus produced, reacts vigorously with certain metals dissolved in contaminated waters forming sparingly soluble metal sulphides (White et al. 2003; Costa and Duarte 2005; Vega-López et al. 2007; Jameson et al. 2010) and, as a result, the concentrations of sulphates and dissolved metals are reduced.

Heavy metals are generally more or less toxic to all types of microorganisms including SRB (Sani et al. 2001; Cabrera et al. 2006). The toxic concentrations of heavy metals to SRB range from a few ppm to as much as 100 ppm (Utgikar et al. 2002). The resistance of SRB to certain metals varies with the species and depends on the concentration of the metal in solution, which can be bacteriostatic or bactericidal. Therefore, a search for metal resistant SRB is much important for the development of efficient bioremediation processes based on the use of these bacteria (Martins et al. 2009).

During the last three decades, a thorough research has been made to decode almost all modes of microbial metal interactions in mesophilic microorganisms for their capable and powerful applications in various bioremediation processes, while the potency of thermophilic microorganisms has largely been unexplored. Thus, thorough and concentrated inquiries on thermophilic microbial metal resistance and their different ways of metal mutual actions can enrich our present knowledge for the improvement of metal bioremediation strategies (Hetzer et al. 2006; Poli et al. 2009; Chatterjee et al. 2010; Sheoran et al. 2010; Ozdemir et al. 2012).

The above highlighted facts reinforce the primary consideration of thermophilic microbial metal interactions at all levels including characterization of inhabitant thermophilic microorganisms and their modes of interactions with metals for designing bioremediation strategies associated with high temperature and other stressful factors. Adequate literature is available on the interaction of mesophilic SRB with toxic metals. However, data on the thermophilic sulphate-reducing bacterial interactions with metals are quite meagre. Pertaining to the context, the present study was designed to probe into the effect haphazardly chosen three toxic metals (Cu, Cr and Ni) on two thermophilic SRB species.

## Materials and methods

### Sampling and chemical characterization

The water samples were recovered from a hot water spring in Chakwal District (32.55°N 72.51°E), Pakistan. The pH and temperature of the water at the time of sampling were 8 and 53 °C, respectively. Multielemental analysis of the collected samples (Table 1) was carried out by proton-induced X-ray emission (PIXE). Then physicochemical parameters including electrical conductivity (EC), carbonates (CO_3_^2−^), bicarbonates (HCO_3_^1−^), chlorides (Cl^1−^) and sulphates (SO_4_^2−^) of the collected samples were measured prior to its further processing for isolation of SRB (Table 1). EC was measured with the help of a digital conductivity meter (UK). CO_3_^2−^, HCO_3_^1−^ and Cl^1−^ were measured following standard protocols (APHA 2005) while SO_4_^2−^ was estimated after Cha et al. (1999).

**Table 1:** Physicochemical and multielemental analyses of the samples

Parameter	Sample 1	Sample 2
Electrical conductivity (Sm^−1^)	19.8	14.6
CO_3_^2−^ (meqL^−1^)	0.00	1.3
HCO_3_^1−^ (meqL^−1^)	3.8	4.2
Cl^1−^ (mgL^−1^)	540	498
SO_4_^2−^ (mgL^−1^)	307	109
Element (Concentration in gKg^−1^)		
As	0.007	0.019
Ba	0.443	0.474
Ca	31.42	27.57
Cd	0.009	0.008
**Cr**	**0.013**	**0.018**
**Cu**	**0.009**	**0.019**
Fe	5.156	4.795
K	19.17	11.62
**Ni**	**0.001**	**0.002**
Pb	0.004	0.013
Sb	0.008	0.009
Se	0.001	0.002
Sn	0.001	0.001
Ti	0.936	0.611
V	0.071	0.043
Zn	0.063	0.137

Metals relevant to this study are shown in bold

### Initial enrichments and isolation of thermophilic SRB

The collected samples were then enriched anaerobically in Postgate B medium. Compositions of different media used in this study are shown in Table 2. All enrichments were made after Hussain and Qazi (2012) by seeding 10 % samples in sterilized serum bottles of 20 mL capacity under anaerobic conditions. The inoculated bottles were incubated at 55 °C till blackening of the medium. SRB growth was confirmed by the formation of black precipitates in addition to production of rotten egg smell of H_2_S. Production of H_2_S was detected by smelling the gas which was obtained from the head space of inoculated vials with the help of sterile insulin syringes. These enrichments were used to isolate pure cultures of the SRB.

**Table 2:** Compositions of various media used in this study

Ingredient	Concentration in gL^−1^			Reference
	Medium B	Medium C	Medium E	
KH_2_PO_4_	0.5	0.5	0.5	Postgate (1984)
NH_4_Cl	1.0	1.0	1.0	
CaSO_4_	1.0	–	–	
MgSO_4_·7H_2_O	2.0	0.06	–	
FeSO_4_·7H_2_O	0.5	–	0.5	
Na_2_SO_4_	–	4.5	1.0	
CaCl_2_·6H_2_O	–	–	1.0	
MgCl_2_·7H_2_O	–	–	2.0	
Sodium lactate	3.5 mL	6.0 mL	3.5	
Yeast extract	1.0	0.25	1.0	
Ascorbic acid	0.1	–	0.1	
Thioglycolic acid	0.1 mL	–	0.1	
Agar	–	–	15.0	

The seed thermophilic SRB were then isolated and pure cultured by repeated application of deep-agar dilution method (dilution-to-extinction method) after Postgate (1984) from water samples using Postgate E medium. pH of the medium was initially adjusted to 7.6 with 1 M NaOH solution. Bacterial pure cultures obtained thus, were maintained in sterile, air-tightened and screw-capped glass bottles filled with Postgate B medium having pH between 7.0 and 7.5 and stored in a dark cupboard at room temperature in Microbial Biotechnology Laboratory, University of the Punjab, Lahore, Pakistan for further experiments.

### Molecular characterization of the bacterial isolates

Characterization of the bacterial isolates at the molecular level involved extraction of total genomic DNA from freshly grown cells of the bacterial isolates in Postgate B medium after Hussain et al. (2014b). 16S rRNA genes were amplified using the universal primers 27f (5′-AGAGTTTGATCMTGGCTCAG-3′) and 1492r (5′-GGTTACCTTGTTACGACTT-3′), which amplify a 1.5 kb fragment. PCR was performed in 50 µL total reaction volume. The reaction mixture used for PCR amplification contained 5 µL of DNA extract, 5 µL of MgCl_2_ (25 mM), 5 µL of dNTPs (1 mM), 5 µL of each primer (5 pmol), 2 U/mL of DNA *Taq* polymerase, 5 µL of 1X *Taq* buffer and 18 µL of DNA free water. PCR amplification was carried out in a thermal cycler (Hamburg 22331, Germany) with a denaturation cycle for 3 min at 94 °C following 35 cycles of denaturation for 30 s at 95 °C, annealing step of 2 min at 60 °C and 1 min extension at 72 °C with a final extension step of 30 min at 72 °C. The PCR products obtained in this way were separated on 1 % (w/v) agarose gel stained with ethidium bromide in TAE buffer by electrophoresis and purified using Gene Purification Kit (Fermentas) following the manufacturer instructions. The amplicons were then got sequenced using Big Dye Terminator v3.1 cycle sequencing ready reactions (Macrogen, Korea) at the DNA Sequencing Facility, Korea. 16S rRNA gene sequences were assembled with phrap (version 0.990319). Homology searches were performed using BLAST (http://www.ncbi.nlm.nih.gov/BLAST/). The 16S rDNA sequences determined in this way were submitted to GenBank for obtaining accession numbers.

### Effect of heavy metals on functioning of the bacteria

Bacterial activity/functioning in terms of sulphate reduction and consequent precipitation of metals (bioprecipitation) was assessed using different concentrations (1, 5, 10 and 15 ppm) of three toxic metals (Cu, Cr and Ni that were prepared using CuSO_4_, Cr_2_(SO_4_)_3_ and NiSO_4_, respectively). All experiments for this purpose were performed in batch and in anaerobic conditions.

### Batch experiments

These were performed in triplicates in sterile serum bottles of 20 mL capacity using modified Postgate C medium. pH of the medium was adjusted to 7.5 ± 0.5. The medium was used with supplementations of predefined concentrations (1, 5, 10 and 15 ppm) of Cu, Cr and Ni independently. Metal solutions were autoclaved separately before adding them to the autoclaved and cooled medium. Sodium lactate was served as growth substrate in these experiments. The inoculum size used was 5 % (v/v) harbouring around 1.7 × 10^6^ C.F.U./mL. pH of the medium was adjusted to 7.5 ± 0.5 for each experiment. Vials with different concentrations (1, 5, 10 and 15 ppm) of these three metals but without inocula served as control. Diffusion of oxygen in inoculated media was prevented by adding a layer of autoclaved and cooled liquid paraffin (about 3–5 mm thick). The inoculated bottles were sealed with fine rubber stoppers and aluminium crimp seals not allowing any air to be trapped in and incubated at 55 °C for 15 days.

### Sampling and analytical methods

Periodically (after every 5 days), 5 mL sample from each incubated vial was withdrawn with the help of a sterile syringe and filtered using a fine quality filter paper (Whatman Cat No. 1001917, UK). Metals and SO_4_^2−^ were analysed for each experiment from the withdrawn samples. For analyses of metals, the samples were acidified with nitric acid and then metals’ concentrations were analysed through atomic absorption spectrophotometer while SO_4_^2−^ was estimated after Cha et al. (1999). After determining un-precipitated concentrations of the metals, bioprecipitation percentages of the metals were also calculated according to the following formula:

$${\text{Bioprecipitation }}(\% ) = \frac{{([{\text{M}}]t = 0 - [{\text{M}}]t = t) \times 100}}{{[{\text{M}}]t = 0}}$$

where [M]*t* = 0 is the dissolved metal concentration at initial time after inoculation and [M]*t* = *t* is the dissolved metal concentration at measure time.

### Statistical analysis

The data were analysed according to Completely Randomized Design (CRD) under factorial arrangement using General Linear Model (GLM) procedures. Means were separated out using Duncan’s Multiple Range (DMR) test with the help of SAS 9.1 for windows (Cary 2002). Differences between means were considered significant at *P* < 0.05.

## Results

### Isolation and molecular characterisation of the bacterial isolates

Two thermophilic SRB species were isolated and pure cultured in this study. BLAST searches of 16S rDNA nucleotide sequences of the bacterial isolates revealed that these bacterial species belonged to the genus *Desulfotomaculum* and the bacteria were identified as *Desulfotomaculum reducens*-HA1 and *Desulfotomaculum hydrothermale*-HA2. The GenBank has allotted accession numbers for these sequences as KF509892 and KF509893, respectively. Both of these two sulphidogenic bacterial species reduced sulphate and precipitated metals in a very close index.

### Effect of heavy metals on functioning of the bacteria

In general, bioprecipitation and sulphate reduction were significant at lower concentrations (1 and 5 ppm) and non-significant at higher concentrations (10 and 15 ppm) of metals. The functioning of bacteria, thus presented an inverse relation with metal concentrations. However, bacterial functioning differed non-significantly between the two species throughout in this study. The overall order of the subject metals’ toxicity to both SRB species appeared as Cu > Cr > Ni. Detailed effects of different concentrations of these metals to the bacteria are described below:

### Effect of copper

**Fig. 1:**
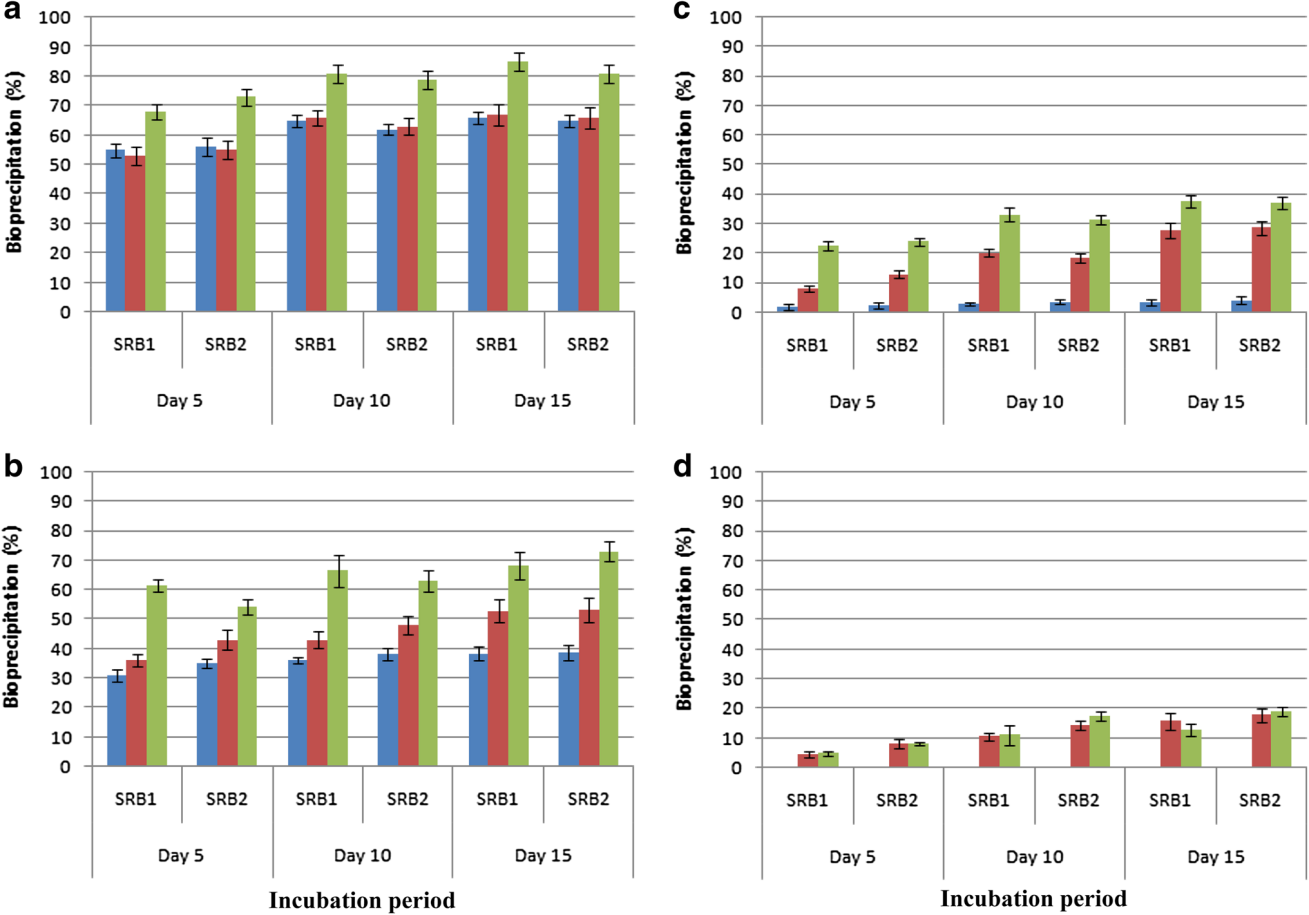
Bacterial profiles of precipitation of metals (Cu, Cr and Ni) under anaerobic conditions at 1 ppm (**a**), 5 ppm (**b**), 10 ppm (**c**) and 15 ppm (**d**) of metals (*blue bar* Cu,* brown bar* Cr and* green bar* Ni) depicting toxic/inhibitory effects of metals. SRB1 and SRB2 represented *Desulfotomaculum reducens*-HA1 and *Desulfotomaculum hydrothermale*-HA2, respectively

Precipitation of Cu could reach maximally to 66, 38.4, 3.3 and 0.27 % at 1, 5, 10 and 15 ppm of Cu, respectively by *Desulfotomaculum reducens*-HA1 after completion of the incubation period. The corresponding figures for precipitation of Cu by *Desulfotomaculum hydrothermale*-HA2 were 65, 38.6, 4.2 and 0.27 at 1, 5, 10 and 15 ppm of Cu, respectively (Fig. 1a–d).

**Fig. 2:**
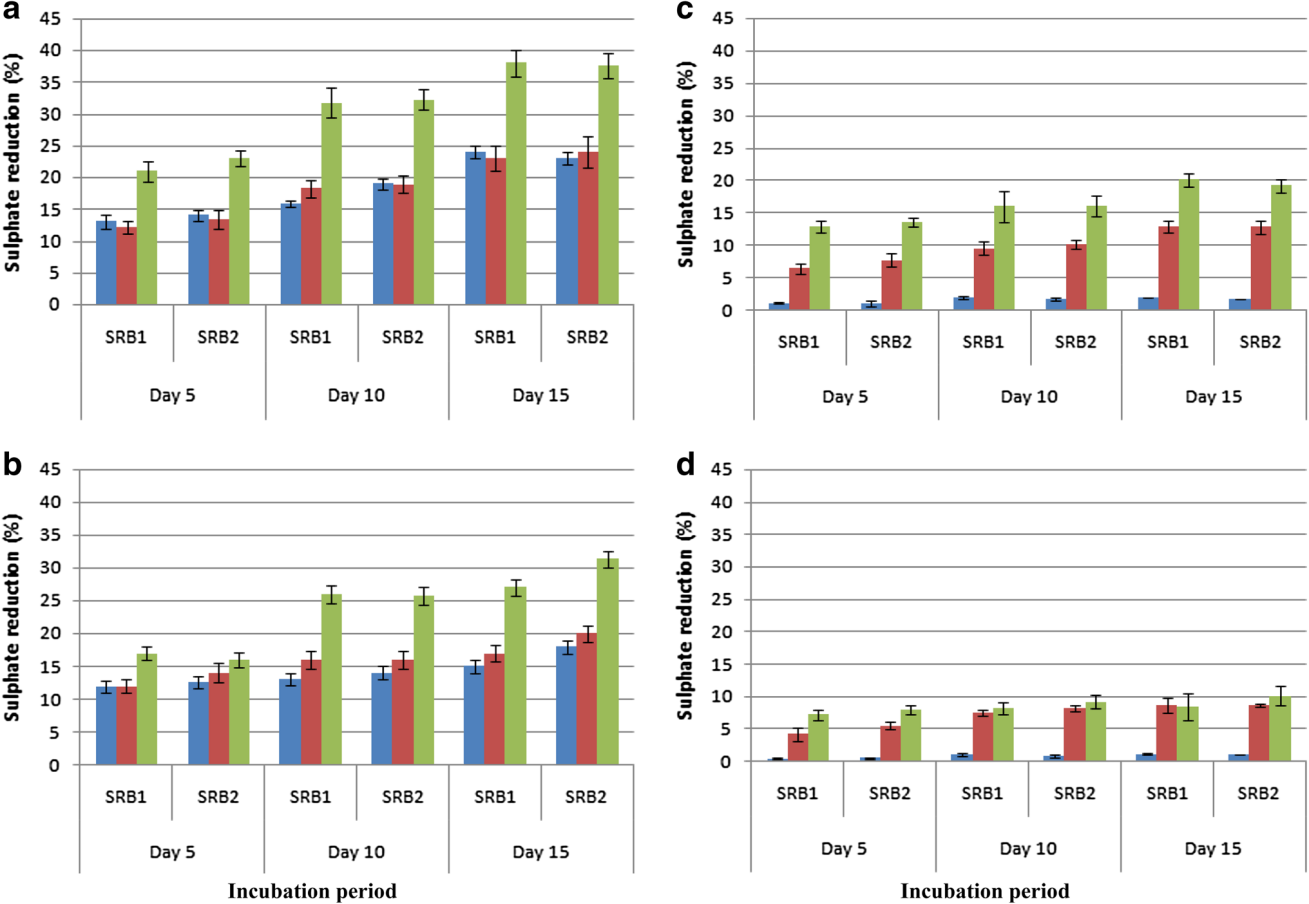
Bacterial profiles of sulphate reductions under anaerobic conditions at 1 ppm (**a**), 5 ppm (**b**), 10 ppm (**c**) and 15 ppm (**d**) of metals (*blue bar* Cu,* brown bar* Cr and* green bar* Ni) depicting toxic/inhibitory effects of metals. SRB1 and SRB2 represented *Desulfotomaculum reducens*-HA1 and *Desulfotomaculum hydrothermale*-HA2, respectively

The metal concentration also influenced the sulphate-reducing capacity of the SRB. An increase in the metal concentration in solution led to decrease in the sulphate reduction rates. For instance, at 1 ppm of Cu the maximum sulphate reduction was recorded to 24 %, at 5 ppm to 15 %, at 10 ppm to 2 % and at 15 ppm to 1.2 % by *Desulfotomaculum reducens*-HA1. The corresponding figures for maximum sulphate reduction by *Desulfotomaculum hydrothermale*-HA2 at 1, 5, 10 and 15 ppm of Cu were 23, 18, 1.7 and 1.1, respectively (Fig. 2a–d).

### Effect of chromium

Chromium toxicity to both bacterial species appeared more or less similar to that of copper at 1 ppm of Cr. However, at relatively higher concentrations (5, 10 and 15 ppm), Cr appeared less toxic than Cu as bioprecipitation and sulphate reduction were much pronounced than those of Cu even at these concentrations. The overall percent precipitation of Cr appeared as 67, 52.8, 27.8 and 15.73 at 1, 5, 10 and 15 ppm of Cr, respectively by *Desulfotomaculum reducens*-HA1. The corresponding figures for precipitation of Cr by *Desulfotomaculum hydrothermale*-HA2 were 66, 53.2, 28.6 and 17.8 at 1, 5, 10 and 15 ppm of Cu, respectively (Fig. 1a–d).

Sulphate-reducing capacity of the both bacterial species decreased gradually as the added concentration of Cr increased. The order of sulphate reduction by the bacteria at different concentrations of Cr appeared as 1 ppm > 5 ppm > 10 ppm > 15 ppm. The overall sulphate-reducing efficiency of *Desulfotomaculum reducens*-HA1 could reach maximally to 23, 17, 12.8, and 8.6 % at 1, 5, 10 and 15 ppm of Cr while the corresponding figures for *Desulfotomaculum hydrothermale*-HA2 were 24, 20, 12.8, and 8.6 at the respective concentrations (Fig. 2a–d).

### Effect of Nickel

In the course of bioprecipitation of Ni, a somewhat different trend was shown by the SRB. Bioprecipitation as well sulphate reduction potentials of the bacteria appeared much better compared to those of copper and chromium even at higher concentrations (10 and 15 ppm). The percent precipitation of Ni reached maximally to 85, 68, 37.7, and 13 at 1, 5, 10, and 15 ppm of Ni, respectively by *Desulfotomaculum reducens*-HA1. The corresponding figures for the precipitation of Ni by *Desulfotomaculum hydrothermale*-HA2 appeared as 81, 73, 37.2, and 18.9 at 1, 5, 10, and 15 ppm of Ni, respectively (Fig. 1a–d).

Sulphate reduction performances were also pronounced at all concentrations of Ni. For instance, at 1 ppm of Ni the maximum sulphate reduction was recorded to 38 %, at 5 ppm to 27 %, at 10 ppm to 20.1 % and at 15 ppm to 8.4 % by *Desulfotomaculum reducens*-HA1. The corresponding figures for maximum sulphate reduction by *Desulfotomaculum hydrothermale*-HA2 at 1, 5, 10 and 15 ppm of Ni were 37.5, 31.3, 19.2 and 10.1 %, respectively (Fig. 2a–d).

In all the experiments dealing with the bioprecipitations of metals (Cu, Cr and Ni), pH of the inoculated media remained in the neutral to slight basic range (7.0 to 8.1) till the end of anaerobic incubation. In control experiments (un-inoculated), metals’ precipitation and sulphate reduction did not occur.

## Discussion

Both bacterial species used in this study for precipitation of the metals showed results in a very narrow range. The changes in sulphate reduction as a function of time with varying amounts of copper appeared variable. A significant decrease in sulphate reduction at 10 ppm of copper indicated that this concentration exerted a significant inhibitory effect on the SRB cultures. This inhibition became more pronounced as the dissolved metal concentration became increased and the assays with higher levels of copper (15 ppm) reduced the ability of SRB to precipitate the metal. These findings were consistent with those of Cabrera et al. (2006) who reported similar results while assessing precipitation of various toxic metals using two mesophilic cultures of SRB (*Desulfovibrio vulgaris* and *Desulfovibrio* sp.). Inhibition of SRB cultures by metals (Cu and Zn) in bioremediation of acid mine drainage was also reported earlier by Utgikar et al. (2002). However, in both of these two cases, mesophilic SRB cultures were employed for the precipitation of metals. Literature on metal precipitation potential of the thermophiles is not available to support or oppose our results of the present study.

Bioprecipitation of copper as well as sulphate reduction occurred maximally at lower concentrations of metal (1 and 5 ppm) and decreased retrogressively as the concentration of the dissolved metal increased (from 10 to 15 ppm). These results were in accordance with the data reported previously (Sani et al. 2001; Utgikar et al. 2003; Cabrera et al. 2006; Azabou et al. 2007; Sheng et al. 2011). Various researchers have proved the stimulatory effects of heavy metals on SRB at lower concentrations and inhibitory (causing a reduction of metabolic activity) or even toxic (causing death) at higher concentrations (Utgikar et al. 2002; Sani et al. 2003). The metal pollutants may inactivate enzymes, denature the proteins and enter in competition with essential cations (Utgikar et al. 2002). But in a study reported earlier by Alexandrino et al. (2011), SRB showed maximum tolerance and activity even at extra ordinary high concentration of copper (more than 500 ppm). However, those bacteria were procured from a highly metal polluted environment where copper concentration was more than 1000 mg/kg (1000 ppm) and therefore, they must have tolerance against much higher concentrations of copper.

Chromium appeared relatively less toxic to the SRB than copper as bioprecipitation and sulphate uptake were even noted at higher concentrations (15 ppm) in this case. This finding was consistent with the previous literature values for toxic concentrations, which were lower for copper than chromium (Cabrera et al. 2006; Ahmadi et al. 2015; Verma et al. 2015). The reason for this difference in cell deactivation could be due to interactions with the bacterial cultures, although further investigations into the exact mechanisms are required to obtain a deeper understanding of this phenomenon as reported earlier by Cabrera et al. (2006).

Negative influence of nickel on the activity of the SRB remained lowest as compared to the cases of Cu and Cr showing remarkable precipitation of the metal at its higher concentration (15 ppm). In a study carried out by Cabrera et al. (2006), nickel concentrations greater than 8.5 ppm exerted a marked inhibitory effect on cultures and this effect was much pronounced for certain SRB strains (*Desulfovibrio vulgaris*). Different isolates, especially those procured from different locations and/or by varying isolation methods do vary in their tolerance levels. This notion necessitates continuous search and isolation of microorganisms employing the suitable selective pressures to search novel microbes as well as to preserve the microbial biodiversity.

An ideal pH of the media used throughout in this study exhibiting neutral to basic range might have further supported precipitation of metals. SRB other than the acidophiles require a pH in the range of 5–8 to grow and metabolize optimally (Willow and Cohen 2003). Beyond this range, generally the rates of microbial sulphate reduction decline and the metal removal capacity is thus reduced. Low pH (<5) inhibits sulphate reduction and increases the solubility of metal sulphide. A pH of 5.5 or higher is however, preferred for efficient treatment in a passive bioreactor (URS 2003).

An interesting point noted in this study was that all the SRB strains showed partial precipitation of these three metals even at lower concentrations (1 and 5 ppm) while having maximum sulphate reduction activity at these concentrations. According to Martins et al. (2009) produced H_2_S easily escapes as a gas during sampling being some of it not accessible to the dissolved metals and thus the precipitation of metals can never be quantitative. This information necessitates optimisation of sulphidogenesis and the metal(s) to be precipitated within tangibly designed bioreactors allowing maximum contact area and time for the H_2_S and metal(s) to react. Further studies must attempt mixtures of different metal ions’ precipitation to determine the order of reactions of different metals with varying concentrations of H_2_S at various physicochemical set ups. Such information might pave diverse metals bioremediation ways necessitating consideration of multiple metals pollutants.

## Conclusions and recommendations

Both thermophilic SRB species obtained and employed in this study showed, in general, significant precipitation of metals at their lower concentrations. Bacteria capable of more metals’ tolerance as well as precipitation potentials be isolated from mining areas to achieve metals bioremediation goal efficiently.

Pure cultures of SRB were employed in the present study for detoxification of metals and sulphates. As at laboratory scale, for determining exact remedial potentials of each microbe, it is necessary to study them in pure cultures. However, the use of mixed cultures is advantageous in providing bacterial consortia that facilitate both the development of reducing conditions as well as utilization of complex nutritive substrates as various researchers have been agreed in this regard. Thus, the implication of mixed cultures of thermophiles for the efficient remediation of metals-loaded wastewaters demand further studies in future.
